# Precision strikes: PSMA-targeted radionuclide therapy in prostate cancer – a narrative review

**DOI:** 10.3389/fonc.2023.1239118

**Published:** 2023-11-16

**Authors:** Paweł Szponar, Piotr Petrasz, Katarzyna Brzeźniakiewicz-Janus, Tomasz Drewa, Piotr Zorga, Jan Adamowicz

**Affiliations:** ^1^ Department of Urology and Urological Oncology, Multidisciplinary Regional Hospital in, Gorzów Wielkopolski, Poland; ^2^ Department and Clinic of Hematology, Oncology and Radiotherapy of the University of Zielona Góra, Multidisciplinary Regional Hospital in, Gorzów Wielkopolski, Poland; ^3^ General and Oncological Urology Clinic, University Hospital No. 1 Dr. Antoni Jurasz, Nicolaus Copernicus University in Toruń, Bydgoszcz, Poland; ^4^ Clinical Department of Nuclear Medicine with a PET/CT Laboratory of the University of Zielona Góra, Multidisciplinary Regional Hospital in, Gorzów Wielkopolski, Poland

**Keywords:** radionuclide, PET-PSMA, 177Luthtet, 225Actinum, CRPC

## Abstract

**Introduction:**

Radio-ligand targeted therapy is a new and promising concept of treatment Castration resistant prostate cancer (CRPC). Only a few radio-pharmaceutics were approved for usage in treating prostate cancer, among the multiple others tested. We aimed to review and summarize the literature on the therapeutic isotopes specific for PSMA.

**Methods:**

We performed a scoping literature review of PubMed from January 1996 to December 2022.

**Results:**

98 publications were selected for inclusion in this review. The studies contained in publications allowed to summarize the data on pharmacokinetics, therapeutic effects, side effects and the medical use of 225Ac and 177Lu radionuclides. The review also presents new research directions for specific PSMA radionuclides.

**Conclusion:**

Radioligand targeted therapy is a new and promising concept where Lu-177-PSMA-617 have promising outcomes in treatment according to standard of care.

## 1 Introduction

In men, prostate cancer (PC) is the most frequent cancer and the third leading cause of death, worldwide. Due to Prostate-Specific Antigen (PSA) testing, PC is diagnosed in early developmental stages when prostatectomy or radiotherapy can be performed with a successfully curative intent. However, in case of metastatic PC, the androgen deprivation therapy is part of the standard care. Nevertheless, in almost all cases, after an average of 2–3 years, the deprivation therapy may generate hormone-resistance, which is defined as the ability of PC to progress even when the testosterone level is at or below the castrate level. Castration-resistant prostate cancer (CRPC) is characterized by poor prognosis, which is reflected in a survival rate <3 years and with limited treatment modalities. In this case, the therapy includes chemotherapy drugs (e.g., docetaxel, cabazitaxel), inhibitors of androgen receptor signaling (ARSI) (e.g., acetate abiraterone, enzalutamide, and apalutamide), inhibitors of polymerase poli-ADP-ribose (PARP), autologous vaccine “Sipuleucel T”, and recently introduced immune checkpoint inhibitors (1–9). Metastatic CRCP (mCRPC) is a terminal disease, so more efficient measures are needed. Radio-ligand targeted therapy is a new and promising concept, where a radioisotope is bound to an antibodies or a small molecules are linked to the radionuclide and these conjugates recognize an antigen expressed on the membrane of the cancer cell.

The development of imaging methods over the years have led to implementation of new ways for PC treatment. Prostate-specific membrane antigen positron emission tomography (PET-PSMA) highlights cancer cells by absorption of radiation from a diagnostic isotope (i.e., 68Ga). That isotope connects with a prostate-specific membrane antigen (PSMA) on prostate gland cells or PC cells. Radionuclides therapy uses the abovementioned mechanism by replacing the diagnostic isotope with a therapeutic isotope. This isotope connects with cancer cell antigens (PSMA) and emits beta (i.e., lutetium-177, yttrium-90) or alpha (actinium-225) radiation. Cancer cells yield ionizing radiation with a minor impact on healthy cells. Only a few radio-pharmaceutics were approved for usage in treating prostate cancer, among the multiple others tested. The most recent radionuclide approved by the FDA for PC treatment is 177LuPSMA-617 (10, 11).

The following review aims to discuss and provide information about therapeutic isotopes specific for PSMA.

## 2 Molecular structure and biological function of the prostate-specific membrane antigen

Glutamine carboxypeptidase II (GCPII), also known as PSMA, has been considered as a potential biomarker of PC, for many years. It is an enzyme coded by FOLH1 gene (folate hydrolase) localized on the short arm of chromosome 11 (11p11-p12) (12, 13). GCPII is identified mainly in prostate epithelium, the proximal tubules of the kidney, the jejunal brush border of the small intestine, and ganglia of the nervous system (14–16). Nevertheless, PSMA undergoes the strongest expression in prostate, where its frequency is >100 times than that in other tissues. In PC cells, PSMA is up-regulated 8–12 times in comparison to non-cancer cells. PSMA expression density on PC cells increases in correlation to the Gleason score for PC (Chang etal., 1999; Elgamal etal., 2000; Minner etal., 2011; Silver etal., 1997) and for CRPC (17). PSMA receptor is capable of internalization of ligands. This process allows the endocytosis of therapeutic isotopes into the intracellular space. Radionuclides that concentrated in cells, close to nearby nucleus, can cause DNA damage and effectively, its apoptosis of the cells by ionizing radiation. Because of this fact, PSMA was chosen as a biomarker in radionuclide therapy (18–20).

## 3 Antigens and “small particles” — ligands bounding with PSMA

Currently used ligands, selective to PSMA, may be divided into antibodies and small particles. The small particles are imaging ligands such as MIP-1095, PSMA-617, and PSMA-I&T. The well-known antibodies are 7E11-C5, J591, and PSMA-TTC.

**Figure 1:**
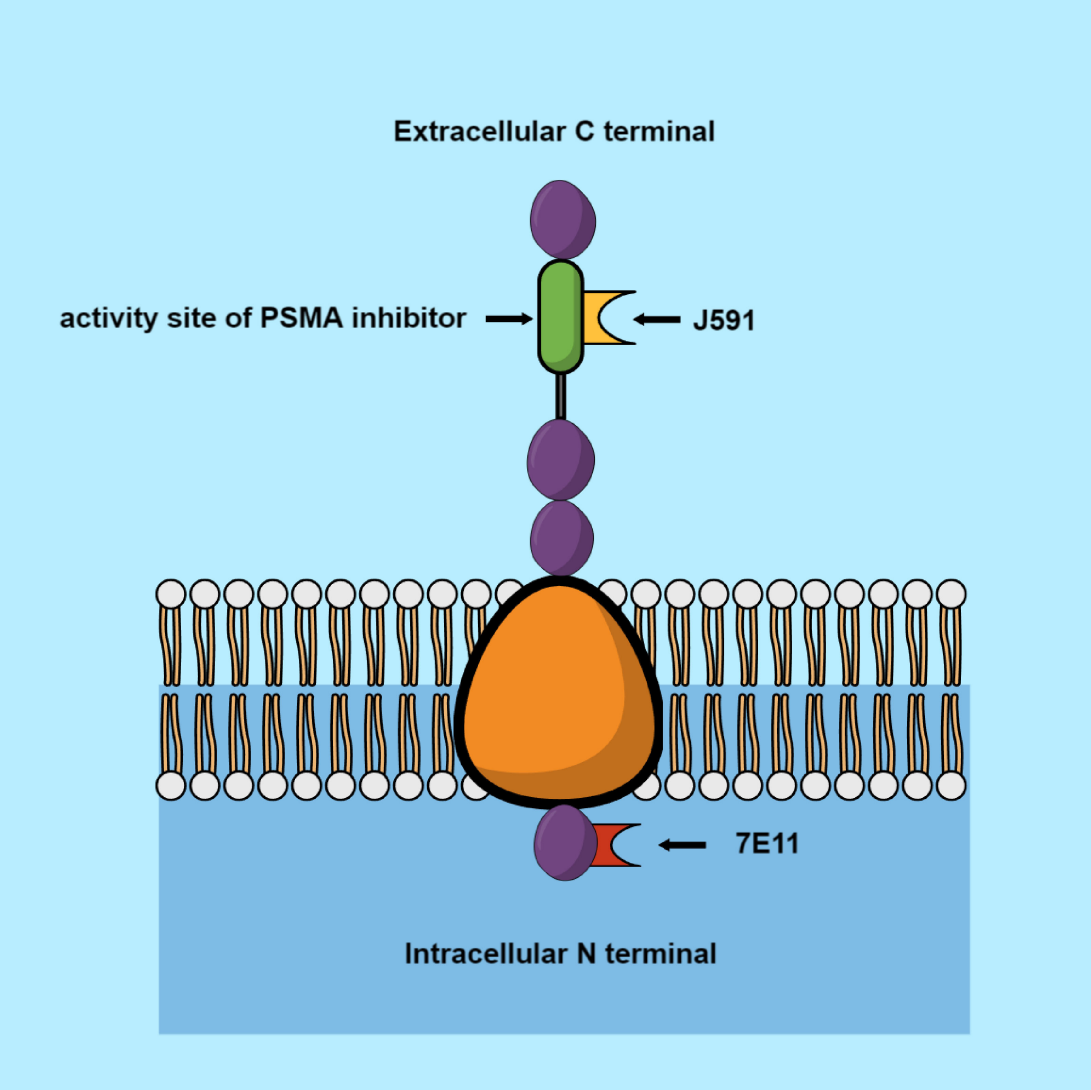
Prostate specific membrane antigen.

The earliest evidence of evaluation of PSMA usefulness for radiotherapy was with 7E11-C5. This molecule targeted an epitope located in the intracellular domain of PSMA. With the help of this antibody, radionuclides of indium-111 and yttrium-90 were made (21, 22). Further studies resulted in the discovery of a monoclonal antibody (J591), in 1997. It was demonstrated that J591 induces PSMA internalization in cancer cells by connection with an external domain (**Figure 1**). The antibody was bound to the radionuclides via a chelating measure dodecane tetra acetic acid (DOTA). The following antibodies were used in these studies: indium-111, yttrium-90, 177Lu, and 225Ac (23–27). Another monoclonal antibody aimed at PSMA is PSMA-TTC (BAY 2315497). TTC is connected with an α-radiation emitter, thorium-227 (227 Th). PSMA-TTC was used in a phase I clinical study (NCT03724747), on patients with developing mCRPC (28). One of the most promising antibodies with a small molecular size (weighing almost two times less than J591, 80kDa vs. 150kDa) is IAB2M. It showed an accelerated plasma clearance, which reduced the amount of radioligand reaching the bone marrow, thereby preventing any severe hematological toxicity. However, the half-life of α-particles posed a problem; they were circulating inside the body, while emitting potentially hazardous ionizing radiation and damaging other organs (29, 30). PSMA-617 is the most frequently used α-particle. This is a small particle, created in Heidelberg (Germany), consisting of a chelator DOTA, which allows it to connect with the radionuclides (31).

The radioactive isotopes mentioned above are applicable in modern PC therapy and imaging.

## 4 Radionuclides used in aimed therapy of prostate cancer

A radionuclide is created by loading radioisotope into the chelator pocket which is bounded to the carrier (a small molecule, a whole antibody or its fragments). They are used in PC treatment after selective binding with PSMA.

The chelator is bound to the carrier (a small molecule, a whole antibody or its fragments), and the radionuclide is loaded into the chelator pocket.

Small molecules are modified PSMA inhibitors in which the structure of the molecule between the linking region and the radioactive metal chelator DOTA has been altered. Consequently, they do not exhibit homology to antibodies and they possess lower affinity in the nanomolar range than antibodies, so they bind less to PSMA in non-cancerous tissues. When combined with a radioactive element, they exert a cytotoxic effect on cancer cells (32, 33).

Noteworthy, small molecule is PSMA-I&T (imaging and therapy), introduced by Weineisen in 2014 as an additional PSMA ligand (34). This molecule has been utilized in both the diagnosis and treatment of advanced prostate cancer. To enhance its lipophilic effect and affinity for PSMA, a peptide linker unit has been incorporated. Furthermore, it has been coupled to 177Lu via DOTAGA.

Another, frequently used radioconjugate, known as MIP-1095, is a urea-based small particle designed for the PSMA radiolabeling with iodine I 131 (I131), and it possesses potential antineoplastic activity. Upon the administration of iodine I 131 MIP-1095, the MIP-1095 moiety selectively targets and binds to the extracellular domain of PSMA, thereby delivering cytotoxic iodine I 131 specifically to PSMA-expressing cancer cells (35).”Antibodies and small particles differ in their molecular structure and function and exhibit differences in kinetics and biodistribution. Accordingly, radionuclides oriented towards PSMA showed higher hematological toxicity when combined with antibodies, whereas when combined with small particles, non-hematological toxicity was observed, including nausea and xerostomia (36, 37). [177Lu] Lu-PSMA and [225Ac] 225Ac-PSMA are the most effective radionuclides available compared to other. Currently known radio pharmaceutics will be presented below.

## 5 Lutetium and actinium: elements which introduced new therapeutic possibilities aimed at PSMA

Iodine I 131 was mentioned in early clinical reports about the usage of radionuclides aimed at PSMA. This element was combined with a small particle MIP-1095, as a PSMA ligand. It showed acceptable pharmacological parameters. Its half-life was short and lasted for 8.02 days. Maximal size of the [I131] I-MIP-1095 was 2.4 micrometers, which caused its rapid internalization by cancer cells. Treatment efficacy was satisfactory, as it led to a 50% decrease in PSA concentration in 60% of the cured patients (38). Currently, it is not used because more efficient radionuclides have been invented. These efficient radionuclides cause less side effects, which will be discussed below. Currently, 225Ac and 177Lu are the most widely used radionuclides in PC treatment.

### 5.1 177 Lutetium

[177Lu] Lu-PSMA-617, also known as Vipivotide Tetraxetan or Pluvictotm are small particle radionuclides approved by FDA for treatment of hormone-resistant PC with metastases. This treatment is suggested for patients who have been treated with inhibitors of androgen receptor and undergone taxan-based therapy (39). The most significant radionuclide characteristics will be presented below.

#### 5.1.1 Pharmacokinetics of Lu-177-PSMA-617

177Lu is an isotope which emits radiation in a wavelength between 1–10 millimeters. The emitted energy from the radiation is 479keV (range, 0.1–10 meV), in total. Lutetium is internalized by cells after PSMA binding with the PSMA-617 ligand. PSMA-617 reaches a short cell penetration range till 670 µm. Radionuclide Lu-PSMA-617 is able to generate ionizing radiation affecting only PC cells with a small impact on other cells due to its characteristics. A seven-day half-life of lutetium ensures an optimal therapeutic effect (40, 41). To initiate cancer cell apoptosis, 96.3 keV of beta radiation should be emitted to these cancer cells, which can be achieved by the binding of the lutetium isotope with at least 1 millimeter volume of tissue. As a result, 177Lu-PSMA-617 is ineffective for treating micro metastases, which are cancer sources <1.2–3.0 millimeters (42).

#### 5.1.2 Clinical trials of 177 lutetium

Lutetium underwent many clinical trials, which resulted in a wide range of radionuclide treatment results. The most important studies on 177Lu were the phase III VISION and phase II TheraP study.

The VISION project measured the effectiveness of 177Lu PSMA-617 treatment on 831 patients with mCRPC. Results of this trial led to the 177Lu PSMA-617 RLT registration by FDA. rPFS (radiologic progression-free survival) and OS (overall survival) were assessed for patients treated with the radioligand 177Lu-PSMA-617, after applying SOC (standard of care) compared to patients who received only SOC. Lutetium treatment extended the rPFS up to 7–8 months, compared to that of patients after SOC who had an rPFS of 3–4 months. OS, after applying lutetium, also extended to 15.3 months, compared to 11.4 months after SOC (43).

In the TheraP study, 177Lu PSMA-617 treatment was compared with cabazitaxel therapy on 200 men with mCRPC. Studies measured the frequency of achieved therapeutic effect, which was defined as a 50% reduction in PSA concentration in blood serum compared to the output levels. The mentioned effect was achieved more frequently in cases treated with 177Lu-PSMA-617 than with cabazitaxel, 66% vs. 37% respectively (44).

German Nuclear Medicine Society conducted one of the largest retrospective clinical trials on 177Lu isotope usage. Results showed that use of 177Lu-PSMA-617 in PC treatment causes a significant decrease in PSA by 50% in the blood serum of 65 patients out of 145. Additionally, QoL (quality of life) rate increased by 60% due to the reduction of pain caused by cancer metastasis to bones (45–48).

“LuPSMA” conducted by Hoffman et al. is a prospective phase II trial. Results showed that lutetium treatment helped in achieving therapeutic efficacy. It was defined as a 50% reduction in PSA concentration in blood serum, which could be acquired in 57% of all cases. The trial included patients with visceral and lymph node cancer metastasis. Lutetium treatment induced an 82% regression rate of metastatic changes (according to Response Evaluation Criteria In Solid Tumors) in this group of patients (49).

OS rate after 177Lu treatment was measured in a few other studies. Violet et al. demonstrate the survival median and OS after lutetium treatment for 13 and 18 months, respectively (48). Gafity and Ahmadzadehfar also observed similar outcomes, after 12 and 13 months of treatment, respectively (10, 50).

Patients treated with 177Lu-PSMA also experienced a recurrence of the cancer. Recurrence median was 6–9 months. In cases where therapeutic effect of lutetium treatment, defined as a 50% reduction in PSA concentration, was achieved, the recurrence time was 8.3 months. In other cases, the recurrence time was 4.2 months. Other trials were conducted, where patients underwent lutetium nuclide treatment cycle. Therapeutic efficacy was achieved in 26–40% of the patients. Almost half of them regained a stable PSA level in approximately 12 months.

In conclusion, the mentioned trials show that 177Lu treatment causes positive therapeutic effects compared to SOC (standard of care) or with cabazitaxel treatment. It effectively extends OS and PFS, along with an increase in QoL.

#### 5.1.3 Toxicity of 177Lu-PSMA-617 according to common terminology criteria for adverse events

Radionuclide treatment may entail some undesired side effects, which must be taken into consideration while making therapeutic decisions. The most frequent side effects of lutetium usage are presented below.

##### 5.1.3.1 Xerostomia

177Lu PSMA-617 accumulated nonspecifically in the salivary glands (51). Salivary glands absorb the largest dosage of radiation among all organs (52). Occurrence of Xerostomia reaches 80–87%, but it is categorized as level 1, according to CTCAE (47). Applying protective therapies for salivary glands such as local cooling, PSMA inhibitors,and injection of botulinum toxin into glands is not significantly effective (53–55). High dosage of monosodium glutamate may decrease radioligand absorption by salivary glands; however, this is pre-clinical data and further research needs to be conducted (56). The lacrimal glands are also capable of radioligand absorption, but only a few cases of dry eye condition were observed.

##### 5.1.3.2 Thrombopenia and anemia

Hematotoxicity of levels 3–4 after 177Lu PSMA-617 radioligand treatment is the most common side effect. Fourth level thrombopenia appears in 10–46% of all cured patients, anemia in 10%, and fourth level neutropenia in 6–26%. The lowest platelet count, as per morphology tests, was observed after 32 days of lutetium treatment. Lymphocytopenia (level 3–4) was one of the most common signs of hematotoxicity, as it was observed in 32% of the cases.

In a German multi-center study, a lesser percentage of anemia, thrombocytopenia, and leukopenia of level 3–4 were observed (10%, 4%, and 3%, respectively) (41). Further research is needed to identify the predictors of hematotoxicity after 177Lu-PSMA-617 radionuclide treatment. Thrombocytopenia seems to be the most common predictor of extensive bone marrow disease and limited marrow reserves, resulting from previous therapies.

Hematotoxicity, which results from the weak permeability of monoclonal antibodies in solids and their large size accompanied by slow elimination from the body, is a critical problem. Appropriate trials using small molecule PSMA inhibitors to limit this effect are in progress (57).

##### 5.1.3.3 Gastroenteritis disorders

Nausea, vomiting, and gastrointestinal disorders usually appear in 50% of the cases, 12 hours after lutetium is given to a patient. It can be caused by an unidentified PSMA expression in the brush border of the proximal part of small intestine, especially duodenum and jejunum. Incidence of this undesired side effect is estimated to be 50%. To avoid this, patients may receive anti-inflammatory and anti-emetic drugs (48, 49, 58–63).

##### 5.1.3.4 Fatigue

Fatigue is the most common side effect reported by patients and its incidence is 50% (64).

##### 5.1.3.5 Nephrotoxicity

PSMA is present in the proximal kidney’s tubules. It leads to nephrotoxicity, as evident in a glomerular filtration rate (GFR) decrease of 11.5 ml after three months of 177Lu treatment (**Table 1**) (58, 59).

**Table 1:** 177Lu-PSMA-617.

Lu-177-PSMA-617	Information
Pharmacokinetics	
Radiation Emission	Lu-177 emits radiation in a wavelength between 1–10 millimeters with energy of 479keV.
Cell Internalization	Lu-177 is internalized by cells after binding with PSMA-617 ligand.
Cell Penetration Range	PSMA-617 reaches short cell penetration range up to 670 µm.
Ionizing Radiation	Lu-177-PSMA-617 generates ionizing radiation, affecting mainly PC cells.
Half-Life	Lutetium has a seven-day half-life for optimal therapeutic effect.
Effective Radiation	96.3 keV beta radiation emission requires binding with ≥1mm tissue volume.
Micro Metastases	Ineffective for micro metastases (<1.2–3.0 millimeters).
Cinical Trial	
VISION Study	Phase III study: Extended rPFS and OS compared to SOC for mCRPC patients.
TheraP Study	Phase II study: Higher therapeutic effect frequency with 177LuPSMA-617.
German Nuclear Medicine Study	Significant PSA reduction, increased QoL observed with 177LuPSMA-617.
LuPSMA Study	Phase II study: Therapeutic efficacy in PSA reduction, regression of metastatic changes.
OS Rate	Various studies show extended OS post 177Lu treatment.
Recurrence	Recurrence median after 177Lu treatment varies based on therapeutic effect.
Toxicity	
Xerostomia	Common side effect: Dry mouth (xerostomia) due to accumulation in salivary glands.
Hematotoxicity	Common side effects include thrombocytopenia, anemia, and neutropenia.
Gastroenteritis Disorders	Nausea, vomiting, and gastrointestinal disorders post-treatment.
Fatigue	Common side effect reported by patients.
Nephrotoxicity	Presence in kidney tubules leads to nephrotoxicity.

#### 5.1.4 Qualification for lutetium treatment

For RLT PSMA treatment, patients should be chosen by a multidisciplinary uro-oncological team, including oncologists, radiotherapists, and urologists, specializing in PC treatment and nuclear medicine specialists.

Prostate Cancer Trials Working Group 3 (PCWG3) shows the qualification criteria for treatment (65): mCRPC with changes described in PET-PSMA, progression after application of at least one cycle of chemotherapy, progression after application of new androgen receptor-targeting agents (ARTA) (abirateron or enzalutamid), progression after application of Radium-223 or without medical indication for usage of this substance, recommendation for 177Lu PSMA-617 RLT in interdisciplinary proceeding.

#### 5.1.5 Other studies

There are many clinical trials being conducted around the world. The preliminary findings of 177Lu-PSMA-617 trials are presented below.

### 5.2 177Lu-DOTAGA-PSMA-I&T

Isotope 177Lu, connected with small particle PSMA-I&T (imaging and therapy), is another currently used radionuclide. PSMA-I&T is used in imaging diagnostics as well as a carrier for therapeutic ligands in PC treatment.

#### 5.2.1 177Lu-PSMA-I&T treatment effects

In a retrospective study conducted by Heck et al., a 30% decrease in PSA concentration was observed in 56% of cases treated with 177Lu-PSMA-I&T (66). Similarly, Baum et al. published a study exhibiting a 50% decrease in 58.9% of all cases. Heck et al. reported a 30% decrease in PSA concentration in 47% of the patients, whereas 38% patients showed more than a 50% decrease. In the trial results, PFS and OS parameters amounted to 4.1 and 12.9 months, respectively (60).

Barber et al. compared results of patients treated with 177Lu-PSMA-I&T and who underwent chemotherapy with those of chemotherapy naive patients. For patients who did not receive chemo-therapy, the median of lifetime PFS and OS amounted to 8.8 and 27.1 months and for patients after chemotherapy, it was 6.0 and 10.7 months, respectively (67).

OS rate in a cohort of chemotherapy-treated patients was longer than in the group where the primary treatment was based on taxanes.

#### 5.2.2 Toxicity of 177Lu-PSMA- I&T

Data showing unwanted side effects of 177Lu-PSMA-I&T treatment are limited. The trial conducted by Heck et al. showed that 9% of the patients suffered from level 3+ anemia, 6% from level 3+ neutropenia, and 4% from level 3+ thrombocytopenia, after 177Lu-PSMA- I&T treatment. Organs which demonstrate the highest amounts of toxicity were the parotid glands and the kidneys (**Table 2**) (60).

**Table 2:** 177Lu-PSMA-I&T.

177Lu-PSMA-I&T	Information
	Isotope 177Lu connected with PSMA-I&T, used for imaging and therapy in PC treatment.
Treatment Effects	
PSA Concentration	
Decrease	- 30% decrease in PSA observed in 56% of cases [Heck et al.]
	- 50% decrease in PSA observed in 58.9% of cases [Baum et al.]
Survival Parameters	- PFS: 4.1 months, OS: 12.9 months [Heck et al.]
	- PFS: 8.8 months (chemotherapy-naive), OS: 27.1 months [Barber et al.]
	- PFS: 6.0 months (post-chemotherapy), OS: 10.7 months [Barber et al.]
Toxicity	
Hematotoxicity	- 9% level 3+ anemia, 6% level 3+ neutropenia, 4% level 3+ thrombocytopenia [Heck et al.]
Organ Toxicity	- Highest toxicity in parotid glands and kidneys [Heck et al.]

### 5.3 177Lu-J591

J591 is an antibody aimed at PSMA which has a long *in vivo* half-life, weak tissue penetration, and long blood half-life compared to the small particles aimed at PSMA (61). Phase I and II trials conducted by Tagawa, Bander et al. proved that a significant PSA decrease was achieved as well as an extension of OS in PC patients after 177Lu-J591 treatment (60, 61). It was shown that there is a decrease in PSA levels, increased survival rate, and higher myelosuppression, depending on the dosage of 177Lu-J591. Also, fractioning of dosages is safer in case of similar accumulated dosages with higher potential efficacies. Tests on the functional dosage of 177Lu-J591 connected with docetaxel were conducted. Patients were administered a standard dose of docetaxel, along with increasing fractionating doses of 177Lu-J591. A study demonstrated the safety of such a connection with early evidences of therapeutic effect, defined as 50% reduction of PSA concentration in blood serum in comparison with entry level (68).

Unwanted side effects reported as follows: 46.8% of the patients suffered from thrombocytopenia level 4 and 25.5% suffered from neutropenia level 4 (**Table 3**) (69).

**Table 3:** 177Lu-J591.

177Lu-J591	Information
	Antibody J591 targets PSMA with long in vivo half-life, weak tissue penetration, and long blood half-life compared to small PSMA-targeting particles.
Treatment Effects	
PSA Decrease and OS Extension	Phase I and II trials by Tagawa, Bander et al.: Significant PSA decrease and OS extension in PC patients after 177Lu-J591 treatment.
	PSA decrease, increased survival, higher myelosuppression observed with 177Lu-J591, dose-dependent.
Dosage Fractioning	Fractioning of dosages is safer for similar accumulated dosages with potential efficacies.
Combination with Docetaxel	Functional dosage of 177Lu-J591 with docetaxel: Safety and early therapeutic effect (50% PSA reduction).
Unwanted Side Effects	
Thrombocytopenia	- 46.8% patients experienced level 4 thrombocytopenia.
Neutropenia	- 25.5% patients experienced level 4 neutropenia.

### 5.4 [225Ac] 225Ac-PSMA-617

From a limited number of α-radiation emitters, only 213Bi, 212Pb, 227Th and 225Ac were evaluated as promising candidates for clinical applications. Studies on these emitters are in progress, but data regarding 225Ac-PSMA-617 is the most impressive and will be the topic of the review survey.

#### 5.4.1 Pharmacokinetics of 225Ac-PSMA-617

After connecting with the binding moiety of PSMA, 225Ac is internalized and works as an “*in vivo* nuclides generator”, emitting 4 α molecules and 2 β molecules after fission (70). Unfortunately, derivative nuclides are not connected with PSMA distribution in tissues and undergo natural biodistribution. It was demonstrated that the early release of free 213Bi along with circulating, tagged antibodies like 225Ac, causes excessive kidney uptake. Accumulation of 90% of maximal tumor uptake may last four days. Its half-life lasts for 9.9 days; it has a short range of radiation in human tissues, which makes it an effective radioisotope. α-emitters have a shorter range of penetration in tissues compared to β-emitters (<0.1mm) and a larger amount of energy (~100keV) (70).

#### 5.4.2 Treatment effects of 225Ac-PSMA-617

Kratochwil et al. analyzed the effectiveness of PSMA 617 in 40 patients with mCRPC (71). Their results showed that PSA was decreased in 87% of cases after 225Ac-PSMA-617 treatment. 63% of the patients achieved more than a 50% PSA decrease in comparison to the entry level. PSA degression was obtained after nine months in the mentioned cases. Based on dosimetry studies, it was determined that 100kBq/kg/cycle is an optimal dosage for use in patients who undergo eight-week breaks between cycles. Effectively, PSA decrease was found to last four months and improved during the next cycles, which are applied every two months (72). Sathekge et al. demonstrated that most of patients after 225Ac-PSMA-617 treatment declared improved quality of life (with bone pains decreased) and minimal side effects (73). Another study from these authors revealed a PSA response in 87% of the patients, more than 70% of who reported a PSA decrease of more than 50%. The median PFS and OS was 15 and 18 months, respectively (74). Yadav et al. reported a >50% PSA decrease in 39% of Irish patients. Median PFS and OS was 12 and 17 months, respectively. A clinical trial at Saar University, Hamburg and in Bad Berka showed that 13% of treated patients come out with almost complete remission (75, 76). Another study showed that 82% of patients reported a 90% decrease in PSA, half of whom obtained undetectable PSA in their serum and remained in remission for next 12 months of therapy (73). Bruchertseifer et al. demonstrated better therapeutic effects when a mixture of radionuclides was applied, including 225Ac-PSMA-617 and 177Lu-PSMA-617 (77).

#### 5.4.3 225Ac-PSMA-617 – side effects

Unwanted side effects of 225Ac treatment were studied by Yadav et al. They did not report toxicity in the bone marrow, whereas some toxicity was observed in kidneys (in 60% of cases, this is primarily caused by nuclides formed from decay of 225Ac, namely 213Bi), but only in a population with known chronic kidney disease (76). Side effects were limited to toxicity of levels 1 and 2 along with xerostomia (in 85% of cases), xerophthalmia, and weight loss in most cases. Very low toxicity has been demonstrated (78). Decrease of toxicity in Bruchertseifer et al. was achieved by “dynamic de-escalation” of dosages (77). The dynamic de-escalation protocol was also used in the Sathekge et al. study where decreased toxicity on the salivary glands was attained (**Table 4**) (75).

**Table 4:** 225Ac-PSMA-617.

225Ac-PSMA-617	Information
	α-radiation emitters considered for clinical use: 213Bi, 212Pb, 227Th, and 225Ac. 225Ac-PSMA-617 studies stand out.
Pharmacokinetics	
PSMA Antibody Connection	After binding with PSMA antibody, 225Ac emits 4 α particles and 2 β particles after fission.
Kidney Uptake	Early release of free 213Bi and tagged antibodies (225Ac) leads to excessive kidney uptake.
Tumor Uptake	90% maximal tumor uptake can last four days.
Half-Life	9.9 days, effective radioisotope with short radiation range (~0.1mm) and higher energy (~100keV).
Treatment Effects	
PSA Decrease	Kratochwil et al.: 87% PSA decrease, 63% >50% PSA decrease, lasting 9 months.
Optimal Dosage	Dosimetry suggests 100kBq/kg/cycle with eight-week breaks between cycles.
PSA Response	PSA decrease lasts 4 months, improves during subsequent cycles (every 2 months).
Quality of Life	Sathekge et al.: Improved quality of life, decreased bone pain, minimal side effects.
PFS and OS	Median PFS 15 months, OS 18 months.
	87% PSA response, >70% >50% PSA decrease, median PFS 12 months, OS 17 months.
	13% complete remission, PSA decrease in 82%, 90% decrease in PSA in 82%, half had undetectable PSA for 12 months.
Combination Therapy	Better therapeutic effects with mixture of radionuclides including 225AcPSMA-617 and 177Lu-PSMA-617.
Side Effects	
Kidney Toxicity	Yadav et al.: Kidney toxicity observed in 60% due to nuclides formed from 225Ac dissolution.
	Chronic kidney disease population showed toxicity.
Other Toxicity	Limited toxicity (levels 1 and 2) with xerostomia (85% cases), xerophthalmia, weight loss.
	Low toxicity reported.
Dynamic De-Escalation	Bruchertseifer et al.: Decreased toxicity with "dynamic de-escalation" of dosages.
	Sathekge et al.: Reduced salivary gland toxicity with dynamic deescalation.

### 5.5 225Ac-J591

Results of phase I trial conducted by Tagawa et al. (NCT03276572) are available. They found that after injection of a single dose of 225Ac-J591 radio-therapeutics 41% out of 22 patients experienced more than a 50% decrease in PSA. One patient developed anemia and level 4 thrombocytopenia, after receiving the dose. Further studies on 225Ac-J591 are in progress (**Table 5**) (79).

**Table 5:** 225Ac-J591.

225Ac-J591	Information
PSA Response	
	41% of 22 patients experienced >50% PSA decrease.
Unwanted Side Effects	
	One patient had anemia and level 4 thrombocytopenia after dose.
Ongoing Studies	
	Further studies on 225Ac-J591 are in progres.

## 6 Comparison of 225Ac and 177Lu

PSMA 225Ac causes more fractures in double-stranded DNA of cancer cells than those caused by 177Lu. This putative mechanism of action should result in better effectiveness. This strategy guarantees therapy optimization in cases with significant bone metastases. Shorter cumulative tissue explosion on β-radiation preserves the intact bone marrow. However, at the same time, toxicity in salivary glands is noticed. Due to this fact, in some centers it is preferred to administer 177Lu- PSMA to patients with soft tissue lesions, whereas 225Ac- PSMA is injected for high volume bone diseases. In preclinical studies, targeted α-therapy was more successful than therapy with β-emitters. [225Ac] Ac-PSMA-617 also demonstrated a high therapeutic efficacy in patients who experienced progression of PC after 177Lu-PSMA-617 treatment, which shows the high potential to concur resistance before β-emitter therapy (71, 72, 74).

## 7 New directions for specific PSMA radionuclides researches

CTT1403 is an organophosphoramidic peptidomimetic, which binds irreversibly with PSMA, allowing radionuclides to be quickly absorbed into the tumor. Because of its albumin binding component, CTT1403 has a long half-life inside blood, which leads to a higher amount of radionuclides build up around the tumor. CTT1403 reduces tumor mass and improves survival rate of animals with tumor cells transplanted (40). CTT1057 demonstrated biodistribution aimed at PSMA, with smaller exposition on kidneys and salivary glands (80). R2 is a PSMA ligand, examined in mice with PC and revealed high tumor uptake when it was marked [177Lu]. R2 is currently tested in PORter phase I/II research, which aims to test safety, tolerance, and dosimetry of radiation of [177Lu] Lu-PSMA-R2 in patients with mCRPC (NCT03490838) (81). Currently, other pre-clinical studies on different radionuclides are taking place: e.g., 149Tb-PSMA-617, 211At-Astatobenzamido-Ureido-Pentanedioic Acid, 213Bi-J591, 213Bi PSMA-I&T vs. JVZ008-nanobody, 225Ac-RPS-074, 227Th-PSMA-IgG, and 212Pb.

## 8 Discussion

CRCP is characterized by poor prognosis that is reflected in a poor survival rate and is a terminal disease. Radioligand targeted therapy is a new and promising concept, where a radioisotope is bound with an antibody or a small molecule ligand. The most recent FDA-approved radionuclide used in PC treatment is 177LuPSMA-617. Lu-177-PSMA-617 have promising outcomes in treatment according to standard of care. It effectively extends OS and PFS and increases the QoL. Radionuclide treatment may entail some undesired side effects. The most frequent side effects are xerostomia and hematotoxicity. From limited number of α emitters, 225Ac is the most impressive. Studies showed that PSA was lower in most cases after 225Ac-PSMA-617 treatment. Side effects were mostly limited to xerostomia, xerophthalmia, and weight loss. The most optimal approach seems to be the use of a combination of different radionuclides for maximum therapeutic effect. β− emitters are most commonly used for RLT, which have a low linear energy transfer (LET). The low deposited energy of β− emitters is only able to yield single-strand breaks of the DNA but can perform this over a longer range, which makes them especially suitable for larger tumors. Getting increasingly interesting for RLT, there are alpha emitters, which have a high LET and lower range; therefore, they are more suitable for smaller tumors and micrometastases. Administering alpha emitters at the onset of disease recurrence, when the maximum dose of beta radiation has been reached using 117Lu, could be a beneficial approach (33). There are multiple new directions for specific PSMA radionuclides research. Radioligand targeted therapy is a new and promising way of treatment of CRCP, which should be the subject of future clinical discussions and research.

## Author contributions

PS and PP contributed to the conception and design of the review, drafting and reviewing of the manuscript, revisions/edits, and a review of references. All authors contributed to the article and approved the submitted version.
